# The Bookworld of Medicine and Science

**Published:** 1904-09-24

**Authors:** 


### The Bookworld of Medicine and Science.

Diseases of the Stomach and their Surgical Treatment. By A. W. Mayo Robson, F.R.C.S., and B. G. A. Moynihan, M.S.Lond., F.R.C.S. Second edition. (London: Baillière, Tindall and Cox. 1904. Pp. 508. Price 15s. net.)

In preparing this edition the authors have considerably amplified, and to a large extent rewritten and rearranged, their former work, greatly to the advantage of the present volume. Two hundred pages have been added, and certain gastric conditions which in the former edition were dealt with in the space of one or two pages have been given chapters to themselves. This is the case with hyperchlorhydria and gastric tetany. In discussing the connection between the former and gastric ulcer, the authors state that they have never failed to discover an ulcer of the stomach or duodenum in an operation case in which excess of hydrochloric had been previously found in the stomach contents. Unfortunately, they omit to mention what they take to be excess of hydrochloric acid, or what tests they apply, so that the statement loses much of its value. With regard to gastric tetany they point out that it is usually a late complication of dilatation of the stomach, and they anticipate that by treating all cases of the latter condition surgically—the only rational treatment—gastric tetany of the lethal type will soon be a forgotten disease. For purposes of reference the book will be essential to everyone who is practically interested in the modern treatment of gastric disorders.

